# Redox-Active Microneedle Platforms for ROS Modulation in Chronic Wound Healing

**DOI:** 10.3390/jfb17100506

**Published:** 2026-10-07

**Authors:** Egemen Umur, Gizem Kaleli-Can, Bugra Ayan

**Affiliations:** 1 Department of Biomedical Engineering, İzmir Demokrasi University, İzmir 35140, Türkiye; 2 BIOMATEN, Center of Excellence in Biomaterials and Tissue Engineering, Middle East Technical University (METU), Ankara 06800, Türkiye; 3 Department of Cardiothoracic Surgery, Stanford University, Stanford, CA 94305, USA

**Keywords:** microneedle, reactive oxygen species, chronic wound healing, hydrogel, biomaterial

## Abstract

Chronic wounds represent a growing clinical and economic burden, and their impaired healing is closely linked to a disrupted redox environment. Reactive oxygen species (ROS) are essential signaling molecules that regulate each phase of wound healing, from hemostasis and inflammation through proliferation and remodeling. ROS at physiological levels regulate antimicrobial defense, angiogenesis and cellular migration. However, the persistent oxidative overload that characterizes chronic wounds sustains inflammation and delays tissue repair. Therefore, restoring redox balance instead of eliminating ROS has emerged as a rational therapeutic strategy. Microneedle (MN) platforms, particularly hydrogel-forming MNs, offer a uniquedelivery approach for this purpose by combining minimally invasive breach of the wound barrier with tunable swelling, high loading capacity, and controlled, localized release of redox-active agents into the wound bed and interstitial fluid. This review examines redox-active MN platforms for ROS modulation in chronic wound management, surveying ROS biology and wound healing physiology, the principal MN geometries and fabrication routes, and three major therapeutic categories: ROS-scavenging systems, ROS-generating systems and material and mechanistic design strategies beyond functional classification. We further discuss current challenges and future directions for translating these platforms toward clinical application. By integrating redox chemistry with MN engineering, hydrogel MN systems provide a versatile route to reestablish redox homeostasis and accelerate healing in chronic wounds. However, redox-active microneedle systems have not yet undergone clinical evaluation. Most efficacy data are derived from experimental animal wound models that do not accurately replicate the complexity of chronic wounds in humans, indicating that clinical translation remains at an early stage.

## 1. Introduction

A wound results from disruption of the epithelial surface and underlying connective tissue, typically caused by physical trauma, thermal injury, or other exogenous factors [1]. Wounds are generally categorized as acute or chronic based on their progression through their healing phase. Acute wounds progress through the normal hemostatic and inflammatory cascades to achieve tissue repair and regeneration within approximately 30 days, usually following surgery, burns, or trauma. Chronic wounds, in contrast, fail to heal within this expected timeframe due to disruption of these sequential phases and the persistence of underlying pathological factors, most notably infection [1,2]. Regardless of the wound type, the healing process involves hemostasis, inflammation, proliferation, and tissue remodeling [3,4].

In the hemostasis phase, platelets aggregate at the wound site to form a clot, driven by activation of the coagulation cascade and the subsequent formation of fibrin fibers [5]. In the inflammation phase, immune cells, primarily neutrophils followed by macrophages, migrate to the wound area and exert phagocytic effects to combat infection and clear cellular debris [6]. Furthermore, these immune cells secrete pro-inflammatory cytokines and growth factors that activate fibroblasts, keratinocytes, and endothelial cells, while also preparing the wound bed for granulation tissue formation [7]. In the proliferation phase, epithelial cells and fibroblasts actively divide and migrate to progressively cover the open wound through the deposition of granulation tissue. This stage is characterized by the assembly and organization of extracellular matrix components, accompanied by the development of new blood vessels. Finally, during the maturation or remodeling phase, the granulation tissue is gradually replaced by collagen fibers, resulting in the formation of mature connective tissue [8].

Reactive oxygen species (ROS) play a crucial role in wound healing. Excessively high ROS levels, arising from either overproduction or impaired detoxification, result in oxidative stress, increased tissue damage, and pathophysiological stalling of the healing process [9,10,11]. Conversely, insufficient ROS levels disrupt the cellular and molecular processes that depend on ROS-mediated signal transduction [1], ultimately contributing to the development of chronic wounds. Therefore, therapeutic strategies capable of precisely modulating ROS levels within the wound microenvironment have emerged as a promising approach for chronic wound management. Consequently, methods capable of restoring this balance, particularly through localized and controlled delivery systems, have become a key focus of contemporary wound care research [12]. Among these emerging strategies, microneedle (MN)-based delivery platforms have attracted considerable attention owing to their capacity for minimally invasive, sustained, and site-specific therapeutic delivery, positioning them as a compelling approach for achieving targeted ROS modulation [13]. Several existing reviews have addressed these areas individually. For example, Joorabloo and Liu focused exclusively on ROS-scavenging nanomaterials for wound healing, without addressing MN-based delivery [14]. Rather et al. reviewed MN-based platforms for the wound healing process as a whole, including immunomodulatory, antimicrobial, and angiogenic functions, but did not treat ROS modulation as a central organizing theme [15]. Haidari et al. specifically reviewed MN-based antimicrobial delivery for wound infection management, a scope distinct from redox modulation [16]. Yun et al. reviewed emerging stimuli-responsive hydrogels, including ROS-responsive systems, for chronic wound healing, but did not include MN-based platforms [17]. Similarly, Jia et al. reviewed smart responsive and controlled-release hydrogels for chronic wound treatment, again without addressing MN-based delivery [18]. None of these reviews has specifically addressed ROS-modulating microneedle platforms, a gap that the present review aims to fill.

This review therefore focuses on redox-active MN platforms as an innovative transdermal delivery strategy for ROS modulation in chronic wound healing. Despite these promising advances, the field is nevertheless mainly preclinical and has limited validation in models that accurately mimic the complexity of chronic wounds in patients.

## 2. ROS in Wound Healing

**Figure 1:**
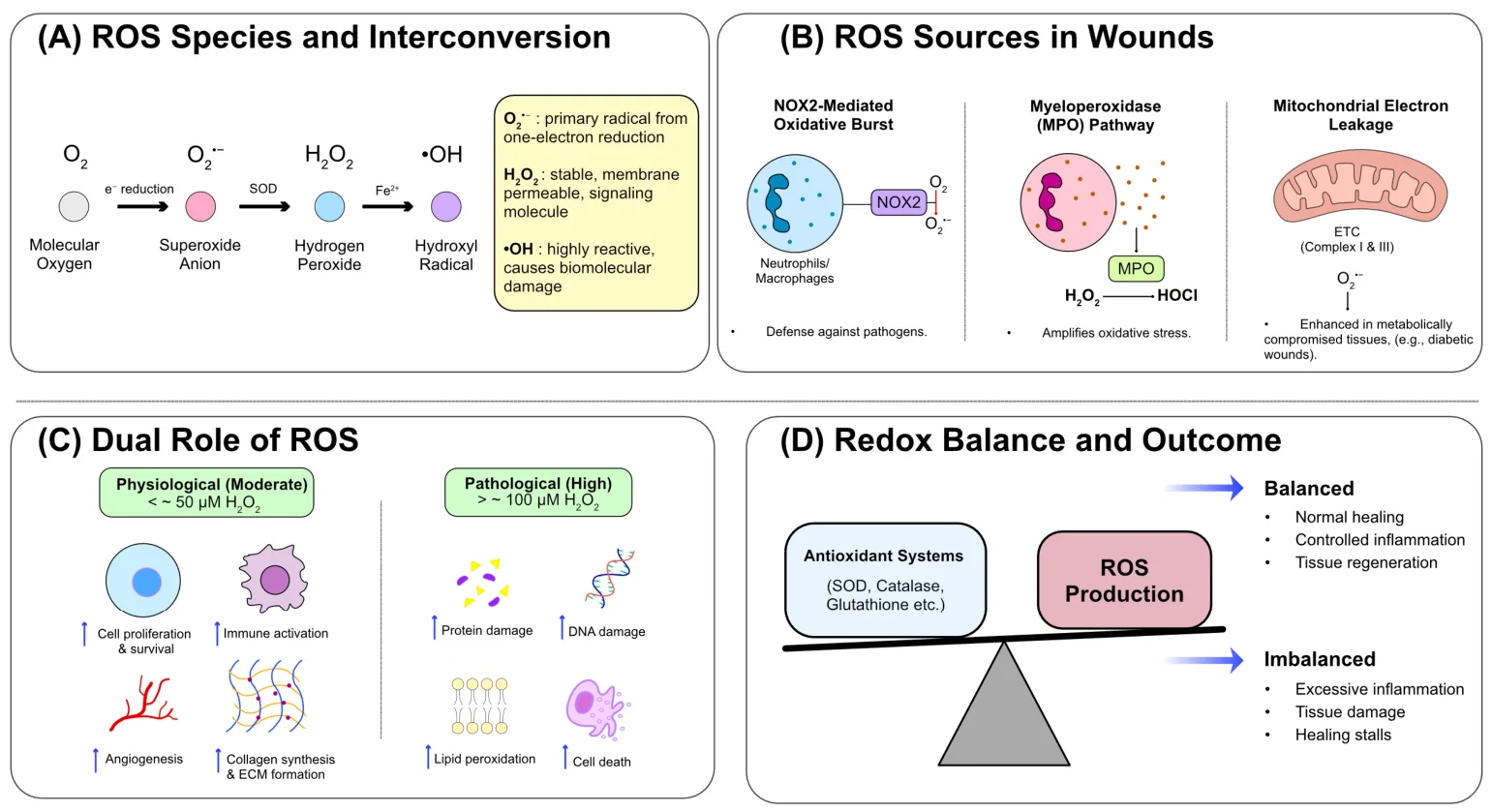
ROS in the wound microenvironment. (**A**) Sequential interconversion of the principal ROS in wound biology: one-electron reduction of molecular oxygen yields O_2_•^−^, SOD catalyzes its dismutation to H_2_O_2_, and Fenton-type reaction with transition metal ions generates •OH. (**B**) The three converging sources of wound ROS: the NOX2-mediated oxidative burst in recruited neutrophils and macrophages, MPO-catalyzed conversion of H_2_O_2_ to HOCl, and electron leakage at complexes I and III of the mitochondrial electron transport chain. (**C**) Concentration-dependent duality of ROS function, supporting proliferation, immune activation, angiogenesis, and matrix assembly at physiological levels while inflicting protein and DNA damage, lipid peroxidation, and cell death at pathological levels. (**D**) Outcome is determined by the balance between ROS production and endogenous antioxidant capacity (SOD, CAT, glutathione). When balanced, healing proceeds normally; when production exceeds scavenging capacity, oxidative stress sustains inflammation and tissue damage and stalls the wound in a non-healing state.

ROS are a chemically diverse group of oxygen-derived molecules consisting of both free radicals, which carry one or more unpaired electrons in their electron shell, and non-radical oxidants [19]. The primary ROS in wound biology include the superoxide anion (O_2_•^−^), hydrogen peroxide (H_2_O_2_), the hydroxyl radical (•OH), and singlet oxygen (^1^O_2_) [20] (Figure 1A). Among these, O_2_•^−^ serves as the primary oxygen radical produced through one-electron oxygen reduction, acting as a precursor to the remaining ROS [21]. Dismutation of O_2_•^−^, catalyzed by superoxide dismutase (SOD), produces the more stable, uncharged H_2_O_2_, which can freely traverse biological membranes and function as a versatile signaling molecule [22]. H_2_O_2_ can subsequently undergo Fenton-type reactions in the presence of transition metal ions to produce the highly reactive •OH [23], which, due to its high electrophilicity and high reactivity, reacts at or near a diffusion-controlled rate with almost all organic biomolecules [24]. In the wound microenvironment, ROS are generated through several converging pathways. The NOX2-mediated release of ROS, also called the oxidative burst, leads to the elimination of invading microorganisms in macrophages and neutrophils recruited to the wound site, where NOX2 catalyzes the one-electron reduction of molecular oxygen to O_2_•^−^ [25]. Concurrently, myeloperoxidase (MPO), stored in azurophilic granules, utilizes H_2_O_2_ as a substrate to catalyze the formation of the chlorinating oxidant HOCl, further amplifying the oxidative stress [26]. Mitochondria represent an additional and constitutive source of ROS, as electron leakage at complexes I and III of the electron transport chain generates large amounts of O_2_•^−^ through a mechanism linked to substrate flux, with this contribution becoming particularly pronounced in metabolically compromised tissues such as diabetic wounds [27,28] (Figure 1B). By contrast, ROS-generating platforms such as CaO_2_ MNs produce oxygen as a byproduct of their hydrolysis reaction, thereby contributing to biofilm disruption and alleviating local hypoxia [29,30]. It should be noted that mitochondrial ROS production was not directly measured in the cited CaO_2_-based MN studies; therefore, the observed benefits of local reoxygenation should not be interpreted as evidence of increased mitochondrial ROS generation in these systems.

While the pathological consequences of ROS excess are well recognized, it is equally important to appreciate that ROS are indispensable signaling molecules at physiological concentrations [31]. A moderate elevation in ROS results in enhanced cell proliferation, survival, and activation of immune responses, whereas high levels lead to cellular damage, including injury to proteins, nucleic acids, and lipids, ultimately resulting in cell death [32] (Figure 1C). In the context of antimicrobial defense, at low concentrations (below ~50 μM), H_2_O_2_ acts as a signaling molecule that activates the redox-sensitive transcription factor NF-κB, enhancing the expression of pro-inflammatory cytokines and antimicrobial peptides. In contrast, higher H_2_O_2_ concentrations (above ~100 μM) can exert direct bactericidal activity through •OH generation [33]. However, since these concentration ranges are mostly derived from controlled experimental conditions and might not accurately reflect H_2_O_2_ levels within the complex and dynamic microenvironment of chronic wounds, they should be interpreted as approximate and case-dependent rather than as fixed therapeutic thresholds. Furthermore, ROS serve as critical second messengers during the proliferative phase of repair; during proliferation, ROS modulate numerous cellular signaling pathways to promote the proliferation, migration, and differentiation of fibroblasts and keratinocytes, and angiogenesis, ultimately facilitating collagen remodeling and extracellular matrix formation [9]. Regarding neovascularization specifically, ROS at physiological concentrations act as second messengers to regulate angiogenesis and vascular repair by activating redox-sensitive kinases and modulating redox-sensitive angiogenic genes such as HIF-1α and VEGF in endothelial cells [34,35]. Studies on scaffold-based bone regeneration also reported M2-oriented macrophage responses alongside improved vascular regeneration [36], indicating that immunomodulation and vascularization may be related regenerative processes in various tissue-repair situations. These diverse physiological roles are conditional upon the maintenance of redox balance. Both the levels and timing of ROS production must be tightly regulated for efficient wound healing: excessive ROS or impaired detoxification leads to oxidative stress, elevated tissue damage, and pathophysiological stalling, whereas insufficient ROS impedes the ROS-dependent signal transduction required for normal cellular responses [8] (Figure 1D). Redox imbalance occurs when ROS levels exceed the capacity of endogenous antioxidants to scavenge them, thereby disrupting the healing process. In chronic wounds, this imbalance can persist, maintaining a prolonged state of oxidative stress and tissue injury [37].

Diabetes is among the most clinically significant reasons for the disruption of redox balance in chronic wounds, as the hyperglycemic milieu amplifies ROS generation through multiple interconnected pathways. Hyperglycemia promotes the formation of advanced glycation end products (AGEs) through the conjugation of glucose to macromolecules; AGEs subsequently activate inflammatory pathways and drive oxidative stress through the generation of ROS by mitochondria rendered dysfunctional under hyperglycemic conditions [38]. Furthermore, sustained AGE-RAGE signaling, together with elevated NF-κB, PKC, and NLRP3 inflammasome activation, promotes ROS generation via NADPH oxidase, progressively overwhelming SOD and catalase (CAT) activity and depleting glutathione stores, thereby exacerbating intracellular redox imbalance [39]. Persistent inflammation is characterized by dysregulation of the immune response, excessive levels of pro-inflammatory cytokines, an altered proteolytic balance with heightened matrix metalloproteinase (MMP) activity, and progressive damage to the extracellular matrix, collectively impairing epithelialization and keratinocyte proliferation [40]. At the cellular level, ROS-mediated activation of NF-κB, together with increased TNF-α signaling, alters MMP secretion from fibroblasts and macrophages, causing excessive extracellular matrix (ECM) degradation. Meanwhile, oxidative damage simultaneously impairs keratinocyte proliferation and migration critical for re-epithelialization, and disrupts angiogenesis through aberrant HIF-1α stabilization and vascular leakage [33]. Collectively, these events sustain a self-perpetuating cycle in which excess ROS drives inflammation, and chronic inflammation in turn amplifies ROS production, preventing the wound from transitioning into the proliferative phase. Given that imbalanced redox conditions and the resulting oxidative damage impair cell proliferation and cause cell death, leading to pathological outcomes including fibrotic scarring and sustained inflammation, MNs capable of dynamically modulating ROS levels within the wound microenvironment have emerged as a compelling therapeutic modality [14,41,42].

### Experimental Wound Models and Their Relevance to Chronic Wound Pathophysiology

The experimental wound models used to evaluate redox-active MN platforms differ substantially in the extent to which they reproduce chronic wound pathophysiology and should therefore not be interpreted as equivalent models of chronic wounds (Table 1) [43,44]. Diabetic wound models reproduce several clinically relevant features of impaired healing, including sustained oxidative stress, dysregulated inflammation, impaired angiogenesis, and delayed re-epithelialization [45,46]. Nevertheless, experimentally induced diabetic wounds remain simplified representations of human diabetic ulcers and do not fully reproduce their prolonged disease history, vascular impairment, neuropathy, or heterogeneous microbial environment [47].

Acute full-thickness excisional wounds in otherwise healthy animals primarily model tissue injury and physiological repair rather than chronic wound pathology [43,48]. Although these models are useful for proof-of-concept assessment of MN biocompatibility, drug release, tissue response, and wound closure, therapeutic efficacy in an acute excisional model should not alone be interpreted as evidence of efficacy in chronic wounds [43,48,49]. Similarly, infection- or biofilm-challenged wounds reproduce the microbial burden and inflammatory component relevant to many hard-to-heal wounds but do not necessarily establish a chronic wound phenotype [50,51]. These models are particularly informative for evaluating antimicrobial MN strategies, including localized ROS generation and biofilm-targeted delivery.

Models combining impaired healing with infection or established biofilm provide a more stringent experimental setting because they incorporate multiple pathological features relevant to chronic wounds [44]. However, even these models remain constrained by species-specific differences in skin architecture, immune responses, and wound closure mechanisms [43,52]. In particular, rodent wounds undergo substantial closure through contraction, whereas human wound repair relies to a greater extent on granulation tissue formation and re-epithelialization, which limits direct translation of wound-closure outcomes from rodents to patients [49,53]. In contrast, rabbit-ear models primarily reproduce hypertrophic scarring and excessive tissue remodeling and should therefore be considered models of fibrosis and scar formation rather than chronic wounds [54,55]. They may provide useful information regarding the effects of redox modulation on remodeling, but their outcomes should not be generalized to chronic wound healing.

Accordingly, the evidence discussed in this review is interpreted according to the pathological feature reproduced by each experimental model rather than treating all wound models as interchangeable evidence of chronic-wound efficacy. This distinction is particularly important for redox-active MNs because the suitability of a model depends on the intended therapeutic mechanism: impaired-healing models are more informative for ROS-scavenging and pro-regenerative strategies, infection- and biofilm-challenged models are more useful for controlled ROS-generating and antimicrobial systems, and scar models are more useful for interventions targeting fibrosis and tissue remodeling.

**Table 1:** Experimental wound models relevant to the evaluation of redox-active MN platforms and their relevance to chronic wound pathophysiology.

Experimental Model	Principal Feature Represented	Main Strength	Key Limitation	Particularly Informative for	Reference(s)
Diabetic impaired-healing wound	Metabolic dysfunction, oxidative stress, persistent inflammation, and delayed repair	Reproduces several major features of impaired healing	Does not fully reproduce the prolonged disease history, neuropathy, vascular impairment, and heterogeneity of human diabetic ulcers	ROS scavenging, redox restoration, angiogenesis, and regenerative responses	[47,56,57]
Acute full-thickness excisional wound	Acute tissue injury and physiological repair	Reproducible and useful for proof-of-concept evaluation	Usually heals efficiently and lacks an intrinsic chronic pathological state	Biocompatibility, release behavior, tissue response, and preliminary wound-closure efficacy	[43,48,58]
Infected/biofilm-challenged wound	Microbial burden and infection-associated inflammation	Enables direct assessment of antimicrobial and antibiofilm activity	Infection alone does not necessarily establish chronic wound pathology	Controlled ROS generation, antimicrobial activity, and biofilm-targeted delivery	[51,59,60]
Impaired-healing + infection/biofilm model	Combined defective repair and microbial challenge	Incorporates multiple pathological features relevant to difficult-to-heal wounds	Remains limited by experimentally induced disease and species-specific healing mechanisms	Multifunctional antimicrobial and redox-regulating MN strategies	[43]
Rabbit-ear scar model	Hypertrophic scarring, fibrosis, and pathological remodeling	Useful for evaluating scar formation and tissue remodeling	Primarily represents fibrosis/remodeling rather than chronic non-healing wounds	Antifibrotic effects and remodeling-associated redox modulation	[54,55,61]

## 3. MN Platforms

MNs are microscale structures, typically ranging from 25 to 2000 μm in both height and width, and are arranged in arrays designed to interface directly with the skin surface [62]. These arrays comprise multiple MNs arranged in a structured pattern, designed to maximize skin contact area and optimize the efficiency of transdermal drug delivery. MN geometry is a critical determinant of performance and they are most commonly fabricated in pyramidical or conical forms. These shapes facilitate efficient skin penetration while minimizing patient discomfort [63]. Based on their delivery mechanism, MNs are classified into five different categories; solid, hollow, coated, dissolving and swelling hydrogel-forming MNs [64]. Each type offers specific functional advantages, making the selection of an appropriate design highly dependent on the intended therapeutic application and the physicochemical properties of the target payload.

### 3.1. MN Geometry

The physical geometry of a MN array governs whether it penetrates the stratum corneum reliably, how deeply its cargo is deposited, and whether the needles survive insertion intact, making geometric optimization a necessity for any MN platform. Four parameters are considered: needle height, tip shape and sharpness, aspect ratio, and array pitch.

Needle height sets the depth of cargo deposition and the tissue compartment that each MN tip reaches. Arrays intended for the dermis are typically fabricated in the several-hundred-micrometer range, which is deep enough to bypass the stratum corneum while remaining short enough to avoid the dermal nerve endings and capillaries that would provoke pain or bleeding [65]. Tip geometry is the primary determinant of penetration efficiency, since a smaller tip radius concentrates the applied force over a smaller contact area to lower the puncture threshold, and both needle shape and tip angle strongly influence the penetration force required [66]. This sharpness requirement is crucial to achieve mechanical robustness, and needle shape is most commonly pyramidal or conical because these forms combine a sharp tip for penetration with a broadening base for structural support [65].

Aspect ratio is defined as the ratio of needle height to base width. A high aspect ratio yields a slender needle that penetrates readily but is prone to bending or buckling failure, whereas a low aspect ratio improves fracture resistance at the cost of penetration efficiency, with increasing aspect ratio and decreasing tip diameter both associated with lower failure forces [65,67]. This exchange is especially consequential for the soft, hydrated polymer matrices favored for redox cargo delivery, which are intrinsically weaker than silicon or metal and therefore more sensitive to aspect ratio-driven buckling [65]. Spacing that is too tight produces a bed-of-nails effect in which the load spreads across so many tips that no individual needle reaches its puncture threshold, and finite-element analysis indicates that closely spaced arrays significantly increase the reaction force and skin strain during the indentation phase before penetration [68,69]. Array density also interacts with mechanical performance, since low-aspect-ratio designs perform better in dense arrays while high-aspect-ratio designs are better suited to sparse configurations [65]. In practice, these parameters are co-optimized rather than set independently since the same slender geometry that improves penetration compromises mechanical integrity, and the balance among them determines whether a given redox-active formulation can be delivered reliably to the chronic wound bed.

### 3.2. MN Classifications and Fabrication

**Figure 2:**
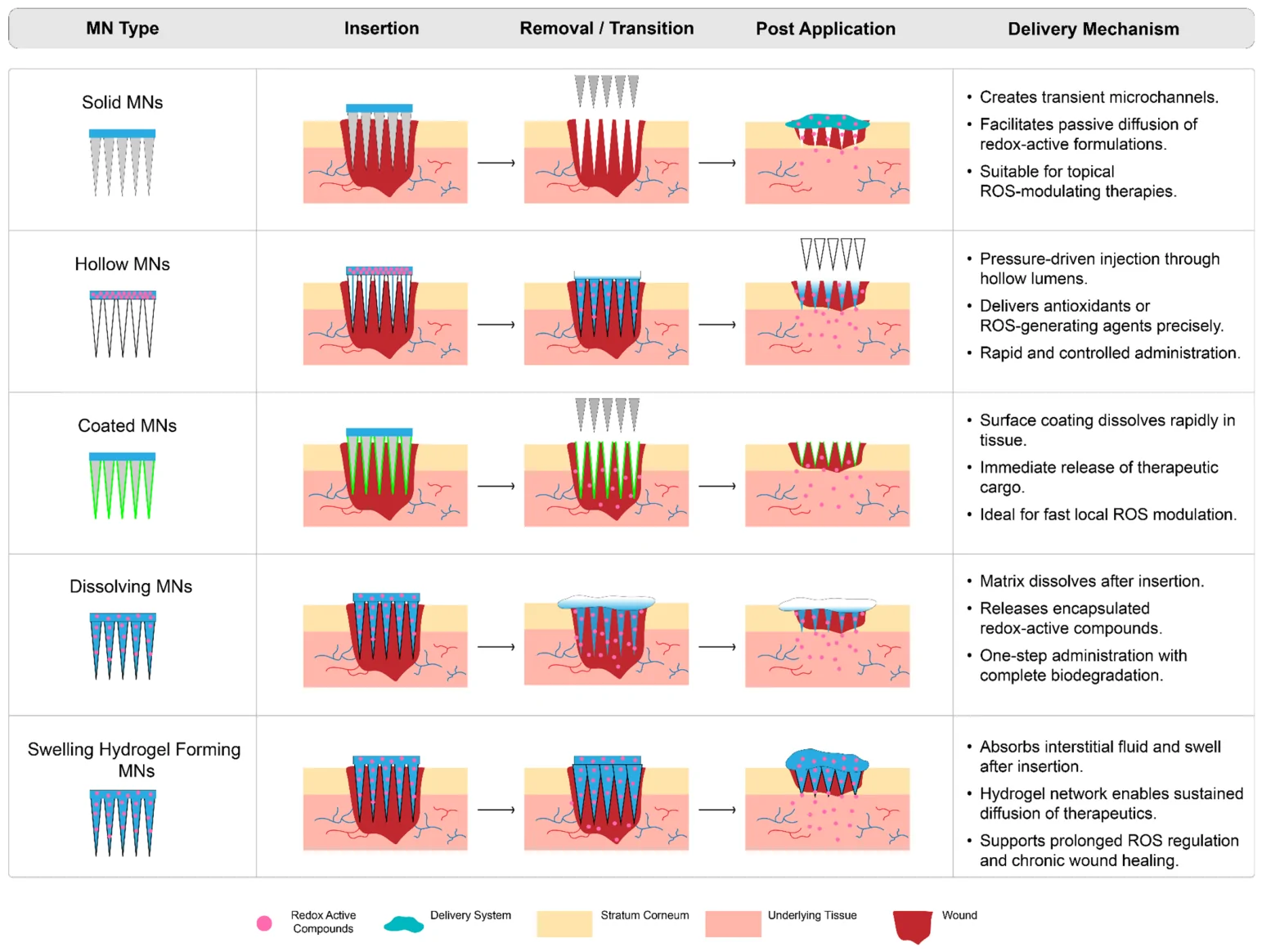
ROS-modulating MN classifications in chronic wound healing.

The structural and material properties of each MN type directly modulate its drug-loading capacity, release kinetics, and compatibility with the wound microenvironment. In the context of chronic wound management, these distinctions carry particular therapeutic significance. The following subsections outline the defining characteristics and delivery mechanisms of each platform type, with emphasis on their suitability for redox-active cargo (Figure 2).

#### 3.2.1. Solid, Hollow and Coated MNs

Solid MNs represent the simplest and most mechanically robust MN type, typically fabricated from silicon, metals such as stainless steel and titanium, or biodegradable polymers such as polylactic acid (PLA) [70]. Their delivery mechanism operates on the “poke-and-patch” principle [71,72] and since the drug is not loaded into the needle itself, solid MNs offer high mechanical integrity and are reusable. However, solid MNs are inherently limited by low cargo specificity and dependence on passive diffusion kinetics, which restricts their utility for large or hydrophilic molecules [73,74]. Because they lack an intrinsic drug reservoir, solid MNs rely entirely on the passive diffusion of externally applied formulations, therebyoffer limited control over the spatially controlled delivery of redox-active cargo in chronic wound settings [75].

Hollow MNs consist of a solid outer shell with a central bore running the length of the needle, enabling the direct injection or continuous infusion of liquid formulations into the dermis [76]. Drug delivery can occur either through passive diffusion or pressure-driven flow through the lumen, enabling direct deposition into the interstitial space upon skin penetration [77,78]. In the context of wound healing, hollow MNs present notable practical limitations: the dense fibrous architecture of the dermis can occlude the needle bore during insertion, reducing flow rates and delivery efficiency, while the requirement to maintain an open lumen imposes strict constraints on wall thickness, increasing their susceptibility to fracture under repeated insertion forces [79,80,81,82,83]. As a result, hollow MNs have found limited application in chronic wound delivery platforms, where the physical fragility of the wound bed and the need for controlled, sustained release favor matrix-based alternatives.

Coated MNs consist of solid MN arrays whose surfaces are functionalized with a thin layer of drug formulation, enabling a rapid “coat-and-poke” delivery mechanism in which the active compound dissolves off the needle surface upon insertion into the skin [84,85]. These systems are typically fabricated from mechanically robust materials such as metals or high-strength polymers, ensuring efficient skin penetration while maintaining structural integrity during insertion [86].

Drug release from coated MNs is characteristically rapid, occurring within seconds to minutes following insertion and this burst-release profile limits their applicability in scenarios that require sustained or controlled release, including chronic wound treatment where prolonged exposure to therapeutic agents is often necessary [87,88]. Moreover, drug-loading capacity is inherently constrained by the limited surface area available for coating [85]. Their reliance on surface-bound drug reservoirs and lack of tunable release kinetics make them less suitable for applications requiring redox modulation in chronic wound environments.

#### 3.2.2. Dissolving MNs and Swelling Hydrogel-Forming MNs (HFMNs)

Dissolving MNs are fabricated from water-soluble and biodegradable polymers that encapsulate the therapeutic payload within the needle matrix itself, enabling a one-step “poke-and-release” delivery mechanism [89,90]. Commonly employed materials include hyaluronic acid, polyvinylpyrrolidone (PVP), polyvinyl alcohol (PVA), carboxymethyl cellulose (CMC), and various sugar-based excipients, all of which dissolve upon insertion into the interstitial fluid of the skin, releasing the loaded cargo in a controlled manner [89,91]. Unlike solid and hollow MNs, dissolving MNs eliminate the need for secondary patches or external reservoirs, thereby minimizing dosing variability and reducing the risk of needle-associated biohazardous waste [90,92].

Recent advances have further demonstrated the incorporation of stimuli-responsive polymers and composite matrices directly into MN platforms, enabling environmentally triggered release profiles in response to ROS levels, pH shifts, or enzymatic activity. These are particularly relevant to chronic wound microenvironments characterized by persistent oxidative stress, alkaline pH, and dysregulated MMP expression. For instance, bilayer MN systems incorporating TSPBA-PVA boronate ester substrates undergo oxidative bond cleavage in response to wound-site H_2_O_2_, releasing antioxidant cargo in a spatiotemporally controlled manner [93], while MN arrays coated with Eudragit S100 exploit the characteristically alkaline pH of chronic wounds (7.3–8.9) to trigger automatic antibiotic release at the wound bed [94]. Enzymatic responsiveness has similarly been achieved through carboxymethyl chitosan/gelatin-based MN tips that undergo disassembly in response to gelatinase secreted by resident wound microbiota, enabling on-demand epidermal growth factor delivery in a diabetic rat wound model in vivo [95]. Ultimately, these stimuli-responsive designs promote dissolving MNs as redox cargo carriers suited for chronic wound ROS microenvironments.

Despite these advantages, dissolving MNs are not without limitations. Their drug-loading capacity is constrained by the small volume of the needle matrix and premature dissolution under high-humidity storage conditions can compromise structural integrity and dosing accuracy [96,97]. Additionally, achieving uniform drug distribution within the MN array remains a manufacturing challenge, particularly for multi-drug or gradient delivery systems [98,99].

HFMNs constitute a distinct MN subclass in which the needle array is fabricated from crosslinked hydrophilic polymer matrices in a dehydrated state, enabling efficient stratum corneum penetration upon application [100]. Following skin insertion, the crosslinked network absorbs dermal interstitial fluid through passive osmotic and capillary driven diffusion, transitioning from a rigid solid to a swollen hydrogel depot that establishes continuous microchannels between the drug reservoir and the surrounding dermal microcirculation [100]. In contrast to dissolving MNs, the covalently or physically crosslinked architecture of HFMNs resists dissolution, forming a persistent hydrogel matrix through which drug molecules diffuse into nearby tissue over an extended period of time [101].

The materials for HFMN fabrication encompass both synthetic and natural-derived polymers, including poly(methyl vinyl ether-co-maleic acid) (PMVE/MA), poly(ethylene glycol) diacrylate (PEGDA), gelatin methacryloyl (GelMA), methacrylated hyaluronic acid (MeHA), silk fibroin, and chitosan, each offering distinct crosslinking chemistries, swelling capacities, degradation profiles, and biocompatibility characteristics suited to specific therapeutic applications [101]. Fabrication is most commonly achieved through UV photopolymerization of liquid precursor solutions cast into polydimethylsiloxane (PDMS) [102]. Critically, crosslinking density can be tuned through adjustment of polymer molecular weight, monomer concentration, and photoinitiator composition, enabling independent modulation of mechanical stiffness, equilibrium swelling ratio, and drug diffusivity within a single formulation framework [103].

In the context of chronic wound management, HFMNs form a sustained hydrogel depot following insertion, enabling prolonged drug retention and controlled release at the target site. Their ability to establish conformal contact with irregular wound beds, combined with their intrinsic biocompatibility, further enhances their suitability for chronic wound applications. Importantly, HFMNs can encapsulate a wide range of therapeutic agents, including redox-active cargos such as nanoparticles (NPs), nanozymes, and metal–organic framework (MOF)-based systems, within a hydrated matrix that can be readily functionalized with stimuli-responsive elements [104,105]. These systems can be engineered to respond to pathological cues such as elevated ROS levels, acidic pH, or aberrant enzymatic activity, enabling adaptive and microenvironment-specific drug release. Collectively, these attributes position HFMNs as a leading architectural framework within the emerging field of redox-active MN systems. Their application in ROS-modulating strategies for chronic wound healing is therefore of particular interest and is discussed in detail in Section 4.

### 3.3. Clinical Rationale and Application Sites for MNs in Chronic Wounds

The rationale for using MNs in chronic wounds differs from that of conventional transdermal applications. In intact skin, MNs are primarily employed to overcome the stratum corneum and facilitate delivery into the viable epidermis or dermis. In an open chronic wound, however, the epidermal barrier may already be partially or completely disrupted, and barrier penetration alone therefore provides an insufficient justification for MN use [106,107]. The potential benefit of MNs in this setting instead depends on their ability to control the localization, retention, and release of therapeutic agents within the wound and surrounding tissue [108,109].

The intended site of MN application is particularly important. MNs applied directly to the wound bed can deposit therapeutic agents within superficial viable tissue, reducing the dependence on diffusion from a formulation placed on the wound surface [106,110]. This may be advantageous in chronic wounds, where heterogeneous tissue composition, wound exudate, necrotic material, and microbial biofilms can affect the retention and distribution of topically administered therapeutics [16,111,112]. Nevertheless, the ability of MNs to penetrate slough, necrotic tissue, or mature biofilms has not been systematically established. Most mechanical characterization of MN insertion has been performed using intact skin or simplified skin substitutes [113,114]. Consequently, insertion depth, mechanical integrity, and drug distribution should be validated under conditions that more closely reproduce the physical characteristics of chronic wound beds.

MNs may also be applied to intact or partially preserved tissue at the wound margins. In this case, penetration of the epidermal barrier remains relevant and may enable local delivery to viable periwound tissue involved in inflammation, angiogenesis, re-epithelialization, and tissue remodeling [106,115]. The distinction between wound-bed and periwound application is therefore important when selecting MN geometry, mechanical properties, and release characteristics.

Conventional topical systems remain advantageous for many wound-care applications. Hydrogel dressings provide extensive wound coverage, maintain a moist environment, and can support sustained delivery, whereas topical gels and sprays can be readily applied to large and irregular wound surfaces [116,117,118,119]. MNs are therefore not necessarily advantageous when surface coverage or moisture management is the principal therapeutic requirement. Their potential value is greater when treatment requires localized deposition, prolonged retention, sequential or compartmentalized delivery of multiple agents, or release triggered by local biochemical conditions [106,120].

These characteristics are particularly relevant to redox-active therapies. As discussed above, the biological effects of ROS depend on their concentration, duration of exposure, and spatial distribution within the wound microenvironment [1,9]. Localization of a ROS-scavenging agent and localization of a ROS-generating antimicrobial agent may therefore produce fundamentally different biological outcomes. In this context, the principal rationale for integrating redox-active therapeutics with MNs is not simply to overcome a physical barrier, but to improve spatial and temporal control over redox modulation within the wound microenvironment.

An important distinction should therefore be made between the therapeutic activity of the redox-active cargo and the contribution of the MN delivery platform itself. Antioxidants, nanozymes, enzymes, photosensitizers, and other redox-active agents may retain their fundamental biological activity when delivered using non-MN formulations. Their incorporation into an MN does not, by itself, demonstrate an MN-specific therapeutic advantage. Such an advantage can be established most directly when an MN formulation is compared with an equivalent non-MN formulation containing the same therapeutic cargo [120,121,122]. In these comparisons, improvements in local retention, tissue distribution, release kinetics, or therapeutic efficacy can more confidently be attributed to the MN architecture rather than to the cargo alone. In the absence of an appropriate non-MN control, improved wound healing or antimicrobial activity demonstrates the efficacy of the combined cargo–MN system, but does not establish the independent contribution of the MN platform. Accordingly, throughout this review, MN-specific benefits are distinguished from the intrinsic biological activity of the incorporated redox-active agents, and conclusions regarding an MN-specific advantage are limited where direct comparative evidence is unavailable. The value of an MN architecture in chronic wound therapy should therefore be judged not simply by its ability to penetrate tissue, but by whether it provides spatial, temporal, or stimulus-dependent control that cannot be achieved as effectively by conventional topical delivery.

## 4. Redox-Active MN Platforms for Chronic Wound Healing

Since the therapeutic goal is to restore redox balance rather than completely suppress or excessively generate ROS, these platforms can be broadly divided into two mechanistically distinct but complementary categories. Importantly, the biological effects of ROS are governed not only by concentration, but also by exposure duration, spatial distribution, and the identity and physiological state of the responding cell population [9]. Transient and spatially restricted ROS signals can participate in antimicrobial defense and cellular signaling, whereas sustained or excessive ROS exposure can damage proteins, lipids, and nucleic acids and impair cells involved in tissue repair [1,123]. Consequently, ROS-scavenging and ROS-generating systems should not be viewed as strictly opposing therapeutic categories, but rather as different strategies for controlling the magnitude, location, and duration of redox activity within the wound microenvironment. ROS-scavenging systems address the oxidative excess of the chronic inflammatory phase, neutralizing pathological ROS to relieve sustained tissue damage and permit the transition into proliferation. ROS-generating systems exploit the antimicrobial and pro-angiogenic properties of controlled ROS elevation, a strategy particularly relevant to infected and biofilm-laden wounds where localized oxidative bursts can help eliminate pathogens while potentially reducing reliance on conventional antibiotics [124]. Beyond these two functional classes, MOF-based MN systems constitute a distinct structural approach in which the porous, high-surface-area framework itself presents redox activity, catalytic capacity, or stimuli-responsive cargo release [125,126]. Finally, stimuli-responsive MN systems represent an integrative design philosophy in which scavenging or generating functions are placed under the control of pathological properties intrinsic to the wound, including elevated H_2_O_2_ and •OH flux, acidic or alkaline pH shifts, and aberrant enzymatic activity, enabling adaptive release that self-regulates in proportion to the local redox burden [127,128]. The following subsections examine each of these categories in turn, with emphasis on the redox chemistry underlying their mechanism of action and the primary research demonstrating their application in chronic wound models (Table 2).

### 4.1. ROS-Scavenging Systems

The chronic wound microenvironment is defined by a sustained accumulation of ROS and inflammatory mediators, which impede the transition of macrophages from the pro-inflammatory M1 to the reparative M2 phenotype, locking the wound in a prolonged inflammatory state [158]. ROS-scavenging platforms are designed to counter this imbalance by lowering pathological ROS toward physiological levels. The agents are grouped into several mechanistically distinct classes, encompassing natural small molecule antioxidants, enzyme mimetic nanozymes, and antioxidant delivery systems [14]. Although the endogenous antioxidant enzymes SOD and CAT maintain redox homeostasis under physiological conditions, their activity is diminished within the diabetic wound microenvironment, aggravating oxidative damage and preserving chronic inflammation [158]. Direct supplementation with native enzymes is further limited by their intrinsic instability, short half-life, as well as the challenges associated with maintaining sufficient enzymatic activity at the wound site. These limitations have motivated the development of engineered scavengers that can provide comparable ROS-neutralizing activity with improved stability, tunability, and cost-effectiveness [159]. The following subsections examine the principal classes of ROS-scavenging cargo integrated into MN platforms: enzyme-mimetic nanozymes, natural antioxidant enzymes, polyphenolic compounds, and antioxidant NPs.

#### 4.1.1. Nanozymes

Nanozymes are nanomaterials that mimic the catalytic behavior of natural antioxidant enzymes, most notably SOD and CAT, offering advantages over their enzymatic counterparts in terms of stability, tunability, and resistance to the harsh oxidative conditions of the chronic wound environment [160,161]. Three metals-based nanozymes are the most studied in this area. Cerium oxide (CeO_2_, ceria) is extensively researched, as the reversible Ce^3+^/Ce^4+^ valence transition provides coupled SOD and CAT-like activity and enables repeated redox cycling without consumption of the catalyst [162,163]. Manganese dioxide (MnO_2_) decomposes the abundant endogenous H_2_O_2_ into O_2_ and thereby coupling ROS depletion with relief of wound bed hypoxia [164,165]. Finally, Prussian blue and its analogs represent a mechanistically distinct class, possessing a broader multi-enzyme profile that spans peroxidase (POD)-, CAT-, and SOD-like activities. Their overall effect can therefore be tuned toward either ROS scavenging or ROS generation depending on the local substrate and intended application [166]. The following studies illustrate how each of these nanozymes has been incorporated into MN platforms for chronic wound management.

Li et al. loaded metformin and CeO_2_ into mesoporous silica NPs (MSN@Met-CeO_2_) and embedded them within a ROS-responsive hydrogel formed into an MN patch with a 600 μm needle height and a 300 μm base diameter, achieving drug penetration to a depth of roughly 130 μm; in a diabetic mouse model, the MSN@Met-CeO_2_ MN was the fastest group to reach 50% wound closure and attained about 95% closure by day 14, whereas the physical-mixture MN and CeO_2_-only MN left 25.13% and 30.21% of the wound area unhealed, respectively [129]. Xuan et al. used lysozyme amyloid fibrils as templates to synthesize ultrafine ceria nanozymes (Lys-AFs-Ceria) with an optimized Ce^3+^/Ce^4+^ ratio and incorporated them, together with glucose oxidase, into a tannic acid hydrogel MN; the templated nanozyme decomposed 89.42% of H_2_O_2_ compared with 56.37% for conventional CeO_2_ NPs, and the assembled MN eliminated 95.3% of O_2_•^−^ and converted 92.97% of H_2_O_2_, driving wound closure beyond 95.71% versus 74.75% in untreated MRSA-infected diabetic wounds at day 14 while achieving 99.72% antibacterial efficiency against *Staphylococcus aureus* [130]. Yan et al. loaded Cu^2+^ onto MnO_2_ nanoflakes through a tannic acid linker (CuTA@MnO_2_) and delivered them by a photothermally controlled MN (needle height 298.56 μm), which cleared nearly 100% of H_2_O_2_ and approximately 90% of O_2_•^−^ within 5 h and provided staged bacterial eradication, oxidant scavenging, and angiogenesis in infected diabetic wounds [131]. Liu et al. constructed a dual-layer MN in which an outer MnO_2_ layer reacts with wound H_2_O_2_ to generate O_2_ and scavenge ROS while an inner layer releases nitric oxide in a sustained manner, promoting diabetic wound repair through combined neurogenesis, angiogenesis, and immune modulation [132]. Prussian blue analogs can be tuned between ROS scavenging and ROS generation; this dual character is exemplified by the ALA@CFP MN of Zhu et al., built from cobalt–iron Prussian blue loaded with 5-aminolevulinic acid, in which POD-like activity deliberately generates •OH from wound H_2_O_2_ to induce bacterial ferroptosis (achieving roughly 100% antibacterial efficiency and 67.4% disruption of mature biofilms) while CAT-like activity concurrently relieves hypoxia, illustrating that Prussian blue nanozyme MNs occupy a mechanistic middle ground between the scavenging systems discussed in this section and the generating systems discussed in Section 4.2 [133]. These comparisons help distinguish the contribution of the individual therapeutic components within the MN formulation; however, they do not independently establish an MN-specific therapeutic advantage over an equivalent non-MN delivery system. The observed outcome should therefore be interpreted primarily as evidence for the efficacy of the integrated cargo–MN formulation.

Collectively, these nanozyme-based MN platforms accelerate wound closure in diabetic infection models; however, their reliance on precise nanostructural control for catalytic performance and the lack of studies beyond mouse/rat models remain unaddressed translational barriers.

#### 4.1.2. Enzyme-Based Systems

In contrast to the nanozyme mimics discussed above, enzyme-based systems employ the native antioxidant enzymes themselves, principally SOD and CAT, which together constitute the endogenous cellular defense against oxidative stress [167]. These two enzymes act in coordination, in which SOD catalyzes the dismutation of O_2_•^−^ into H_2_O_2_ and CAT subsequently decomposes H_2_O_2_ into H_2_O and O_2_, thereby neutralizing pathological ROS while simultaneously relieving the hypoxia that characterizes the chronic wound bed [167,168]. Their principal advantage over synthetic nanozymes lies in their high substrate specificity and near diffusion limited catalytic efficiency. This advantage, however, is offset by well-recognized liabilities since native enzymes are prone to denaturation, proteolytic degradation, and rapid loss of activity in the protease-rich environment of a chronic wound [160,169]. The following studies illustrate how enzyme-based systems have been incorporated into MN platforms, most commonly by pairing CAT with a peroxide-generating source to establish a self-sustaining oxygen supply within the wound.

A representative example was reported by Sun et al. who built a silk fibroin methacryloyl (SilMA) MN patch with tips loaded with CaO_2_ and CAT and a base coated with silver NPs. CaO_2_ reacts with water to form H_2_O_2_, which CAT decomposes into oxygen and water. CAT free formulations produced the highest ROS and severe fibroblast cytotoxicity, whereas the CAT containing patch preserved viability. The construct sustained oxygen release for seven days, increased the M2 macrophage fraction, and lowered IL-1beta and TNF-alpha in a diabetic mouse model [134]. Barnum et al. further fabricated a shelf-stable GelMA array co-loaded with CaO_2_ and 1% CAT. The optimized array raised local oxygen tension by about 22 mmHg, shifting tissue from the ischemic range (5 to 30 mmHg) into the healthy range (30 to 60 mmHg), and reversed the chronic hypoxia [135]. The CAT-(MnSH)x@PMB system developed by Cai et al. instead uses CAT as a biological template to biomineralize a manganese nanozyme, onto which polymyxin B is electrostatically bound, and additionally releases hydrogen sulfide for antioxidant and anti-inflammatory activity. Delivered on a soluble PVA and hydroxyethyl methacrylate substrate, it upregulated M2 macrophages, reduced TNF-alpha, and promoted angiogenesis and nerve regeneration [136].

Across these three platforms, coupling CAT with a peroxide-generating source (CaO_2_) proves a consistent strategy for simultaneously scavenging ROS and relieving wound hypoxia. Furthermore, addition of polymyxin B illustrates a shift toward multi-cargo designs which broadens the therapeutic scope but the long-term immunological tolerability of protein-based MN cargo remains an open question.

#### 4.1.3. Polyphenolic Compounds

Naturally derived polyphenols represent a widely studied class of small-molecule antioxidants for MN delivery, with curcumin, tannic acid, and gallic acid among the most extensively studied [170]. Their ROS-scavenging activity is primarily attributed to their abundant phenolic hydroxyl groups, which can neutralize free radicals through hydrogen-atom and electron donation. In tannic acid, the catechol and pyrogallol moieties further contribute to its antioxidant activity, while these functional groups also provide complementary anti-inflammatory, antibacterial, tissue-adhesive, and metal-chelating properties [171,172]. However, their principal liability is poor aqueous solubility and low bioavailability, most acutely for curcumin, which restricts topical efficacy and motivates encapsulation [173]. MN platforms address this by carrying the polyphenol, often pre-formulated within microparticles or nanocomposites, across the stratum corneum for localized, sustained release into the dermal compartment.

Curcumin has most commonly been delivered from a protective secondary carrier embedded within the needle matrix. Zhang et al. constructed a double-layered GelMA MN (545 μm needle height and 384 μm base diameter) with electrosprayed curcumin-loaded poly lactic-co-glycolic acid (PLGA) microparticles clustered in the tips and zinc oxide NPs in the base, with the latter forming a physical barrier with antibacterial activity against both *S. aureus* and *Escherichia coli*. The microparticle reservoir converted a 28.9% burst within the first 24 h into a sustained release reaching 62.2% by day 7 and continuing over 20 days. The curcumin-containing groups scavenged 81.6% and 78.3% of DPPH radicals versus 24.3% for the zinc-oxide-only group, accelerated fibroblast scratch closure to 89.7% at 48 h compared to 58.5% for the untreated control, and, downregulated alpha-SMA and type I collagen, reducing hypertrophic scarring in a rabbit-ear model [137]. A stimuli-responsive iteration was reported by Zheng et al., who reinforced a hydrogel MN with niobium carbide (Nb2C) nanosheets and curcumin, in which curcumin acts as the primary extracellular ROS scavenger while near-infrared irradiation of the embedded Nb2C triggers on-demand curcumin release and adds POD mimetic and photothermal antimicrobial activities, establishing a dual ROS-scavenging and antibacterial platform for diabetic wounds [138].

Tannic acid and gallic acid have been used as components of metal phenolic or MOF networks. Li et al. incorporated MXene-tannic acid/iron (MXene-TA/Fe) nanosheets, in which tannic acid coordinates iron within a metal–phenolic network on the MXene surface, into a GelMA/PEGDA MN; under mild near-infrared irradiation, the patch reached radical-scavenging capacities of roughly 70% for DPPH and 85.2% for ABTS, showing pH-dependent activity that was optimal under weakly alkaline conditions (pH 7.5 to 9.5), and reprogrammed diabetic-associated macrophages for deep-wound infection therapy [139]. In a related MOF design by Yin et al., the dual-layer Mg-MOF MN loaded gallic acid-based magnesium organic frameworks (Mg-MOFs, roughly 6 μm microspheres) within a poly(gamma-glutamic acid) tip layer with a 500 μm needle height and 200 μm base diameter, from which Mg^2+^ and gallic acid, a ROS-scavenging polyphenol, were released under the acidic wound microenvironment, while a graphene oxide/silver backing layer supplied antibacterial function against *S. aureus*, *E. coli*, and *Pseudomonas aeruginosa*. The dissolving tips released 81.4% of their Mg-MOF within 30 min, and DPPH scavenging rose with Mg-MOF concentration to a maximum of 73% at 60 μg/mL. Under 3 mM H_2_O_2_ oxidative challenge, fibroblast viability was increased from 49% in the untreated group to 74%, 94%, and 97% at 30 μg/mL, 60 μg/mL, and the full patch extract, respectively, with a corresponding drop in intracellular ROS. In diabetic mouse model, the full MN-MOF-GO-Ag patch reduced the residual wound area to 12% by day 17 compared to 74% for the untreated control and produced the smallest granulation tissue width and the highest CD31 capillary density among all groups, reflecting the synergy of gallic-acid antioxidation, Mg^2+^-driven angiogenesis, and GO-Ag antibacterial action [140].

While curcumin, gallic acid and tannic acid all demonstrate the antioxidant versatility of phenolic compounds when delivered via MN platforms, the diversity of the source material also means that their release kinetics and catalytic behavior are governed by distinct chemistries. A shared limitation across all three compounds is their long-term stability; phenolic compounds are inherently prone to oxidative degradation under exposure to elevated pH, oxygen and light, which raises practical concerns for MN shelf life and storage [174].

#### 4.1.4. Antioxidant NPs

The final class of ROS-scavenging cargo comprises antioxidant NPs whose radical-neutralizing capacity is an intrinsic property of the material, with melanin-based and selenium-based NPs representing two major families. Melanin NPs and melanin-mimetic materials contain catechol- and indole-rich polyphenolic surfaces that quench a broad range of radicals through sequential electron and hydrogen-atom donation. In addition to their antioxidant activity, these materials exhibit strong near-infrared absorption, enabling photothermal antibacterial effects, metal chelation, and good biocompatibility [175,176,177,178]. Selenium-based NPs instead exploit the redox chemistry of selenium itself, which cycles between oxidation states to mimic the endogenous glutathione peroxidase (GPx) pathway [179,180,181]. Integrating these NPs into MN arrays delivers durable, catalytically or stoichiometrically self-sustaining antioxidant capacity directly into the dermal compartment while enabling adjunct photothermal or stimuli-responsive functions.

Melanin NPs have been incorporated into MN platforms primarily where a combined antioxidant and photothermal function is desired. Lei et al. functionalized a hyaluronic acid MN (600 μm needle height and 300 μm base diameter), with biomineralized cuttlefish-ink melanin NPs (186 nm cores) encapsulated in an amorphous silica shell (CINP@SiO_2_-HA MN). The melanin core provided concentration-dependent near-infrared photothermal heating, reaching around 54 to 73 degrees at 0.15 to 0.20 W/cm^2^, that delivered about 95% antibacterial efficiency against *S. aureus*, while the silica shell released bioactive silicate ions over 15 days to upregulate angiogenic genes. In an infected full-thickness wound model, the CINP@SiO_2_-HA MN under irradiation lowered wound ROS by 78%, raised wound closure to 93% by day 15 compared to 60% for untreated controls, and increased CD31 micro-vessel density by 70%. These findings indicate that the single patch system combined ROS scavenging, photothermal antibacterial activity and enhanced revascularization [141]. A melanin-mimetic strategy was reported by Ma et al., who encapsulated polydopamine NPs (54 nm) in the methacrylated hyaluronic acid shell of a core–shell MN tip while housing iron-loaded mesenchymal-stem-cell-derived nanovesicles in the inner core. The gradually degrading shell sustainably released the polydopamine NPs, which reduced H_2_O_2_-induced intracellular ROS in a dose-dependent manner (maximal at 80 μg/mL), while the combined platform increased the M2 macrophage fraction to 40.1% and lowered the M1 fraction to 6.4%; in a diabetic rat wound, it reduced the residual wound area to 11.8% by day 12 compared to 29.4% for the control, with the highest collagen deposition and CD31 vessel density among all groups [142].

Selenium-based NPs have been engineered to combine intrinsic radical scavenging with sequential therapeutic release. Zhang et al. constructed a dissolvable dual-layer GelMA MN loaded with selenium-doped carbon quantum dots (Se-CQDs) synthesized from L-selenocystine in the outer layer and the flavonoid astilbin in the inner layer. The bilayer geometry established a sequential action in which the outer-layer Se-CQDs first cleared excess ROS to relieve oxidative stress, after which the inner-layer astilbin exerted anti-inflammatory and pro-angiogenic effects by promoting M2 polarization. As a result, in streptozotocin-induced diabetic mice, the full Se-CQDs/AST patch produced the fastest wound closure among all groups, with nearly complete closure by day 14, together with the densest collagen deposition and the highest CD31-positive microvessel density [143].

The intrinsic antioxidant properties of melanin- and selenium-based NPs simplify the formulation but also remove the tunability that pairing strategies (e.g., CAT with CaO_2_) afford. For instance, the antibacterial effect in Lei et al.’s CINP@SiO_2_-HA MN depends entirely on external NIR irradiation held within a narrow temperature window. Furthermore, selenium is well documented to shift from antioxidant to pro-oxidant and cytotoxic at only moderately elevated doses [182,183]. Finally, a translational question specific to this class is the long-term biodistribution and clearance of these nanoparticle cores.

### 4.2. ROS-Generating Systems

ROS-generating MN systems elevate the local ROS concentration to exploit its antimicrobial and pro-angiogenic effects, a strategy suited to infected, biofilm-laden, or hypoxic chronic wounds where conventional antibiotics struggle to penetrate. Since sustained bactericidal ROS flux is required to overcome biofilm resistance, these platforms rely on chemistries capable of producing reactive species continuously or on demand once released into the wound bed [184,185]. This ROS-generating approach can be broadly divided into five categories: solid peroxide compounds that hydrolyze to generate H_2_O_2_ and downstream ROS, photosensitizers that generate ^1^O_2_ and other reactive species upon light activation, sonodynamic agents that achieve the analogous effect under ultrasound, chemodynamic agents that catalyze Fenton or Fenton-like reactions to convert endogenous H_2_O_2_ into highly reactive •OH, and pro-oxidant NPs whose intrinsic semiconductor or catalytic properties promote ROS generation. A central challenge for ROS-generating systems is therefore to achieve sufficient oxidative activity for antimicrobial action without exposing surrounding host tissue to prolonged or excessive ROS levels. This balance can be maintained through spatial confinement of ROS-generating agents, control over dose and release kinetics, externally triggered activation that limits ROS production to defined treatment periods, or coupling ROS generation to pathological features of the wound microenvironment, such as elevated glucose or local pH changes [186,187,188]. Nevertheless, the therapeutic window is likely to vary with ROS species, exposure duration, microbial susceptibility, and the sensitivity of surrounding host cells, emphasizing the need for quantitative evaluation of both antimicrobial efficacy and host–cell and tissue toxicity [186,189]. The following subsections examine each class in turn.

#### 4.2.1. Peroxide-Based Compounds

Solid metal peroxides, principally CaO_2_ and, less commonly in MN formulations, MgO_2_, constitute the most direct route to controlled ROS generation. Upon contact with the aqueous wound environment, these compounds hydrolyze to release H_2_O_2_ and molecular oxygen, with the release rate influenced by particle size and matrix encapsulation. The resulting H_2_O_2_ flux can act directly as a bactericidal and biofilm-disrupting agent [190]. Peroxide-based MNs therefore represent the generating side of the scavenging-generating divide, with their primary therapeutic function being to maintain localized oxidative activity against pathogens rather than neutralize ROS. At the same time, the hydrolysis reaction can alleviate wound-bed hypoxia by releasing oxygen [186].

**Figure 3:**
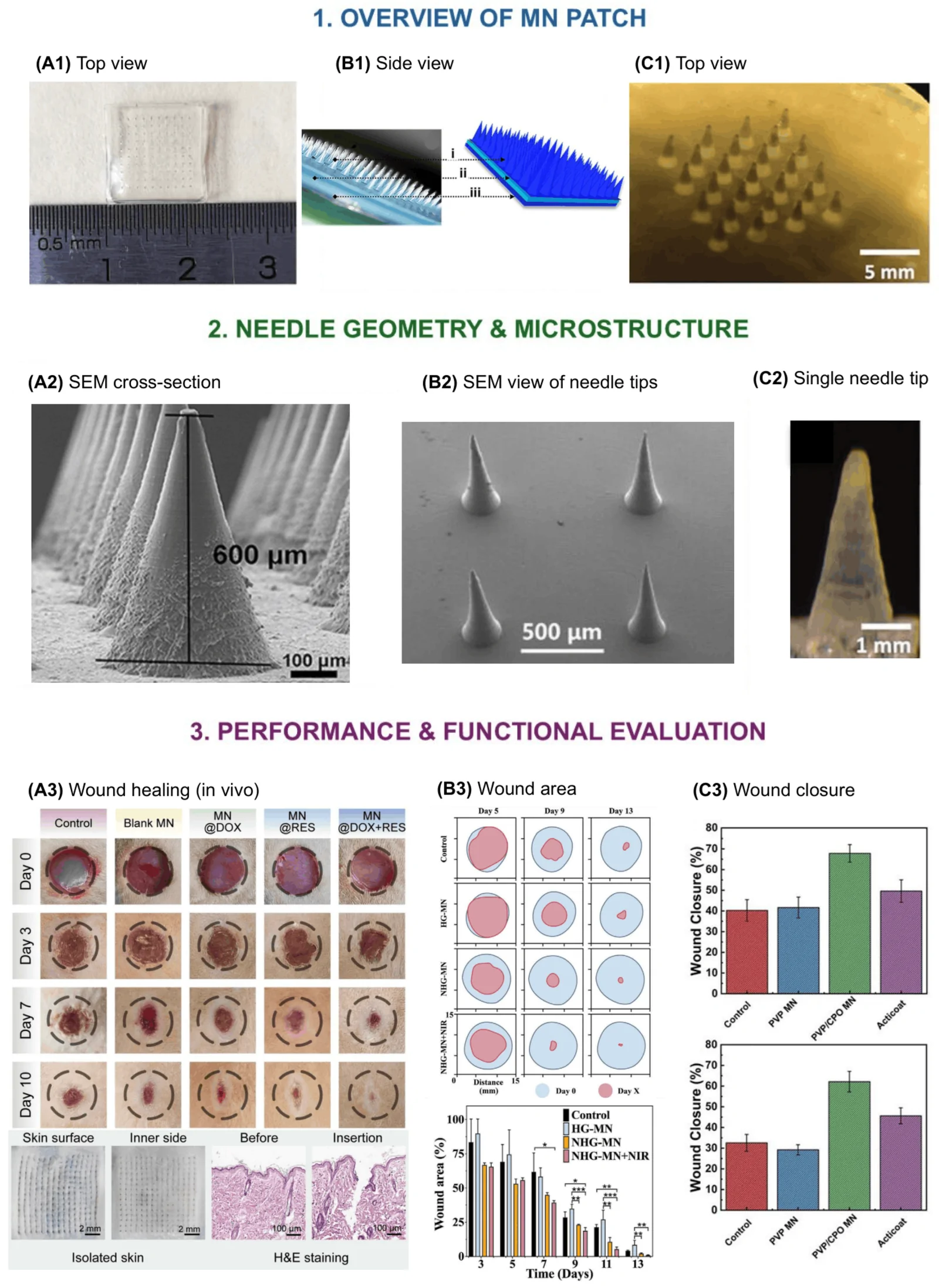
Overview and characterization of MN patches. (1) Overview of MN patch: (**A1**) adapted from Lin et al. [152], (**B1**) adapted from Xin et al. [156], (**C1**) adapted from Krishnakumar et al. [29]. (2) Needle geometry and microstructure: (**A2**) adapted from Xin et al. [156], (**B2**) adapted from Lin et al. [152], (**C2**) adapted from Krishnakumar et al. [29]. (3) Performance and functional evaluation of MN patches: (**A3**) adapted with permission from Ref. [154], (**B3**) adapted with permission from Ref. [125] (statistical significance is indicated as follows: * *p* < 0.05, ** *p* < 0.01, and *** *p* < 0.001), (**C3**) Adapted with permission from Ref. [29].

Krishnakumar et al. developed a water-dissolvable PVP MN loaded with 10 wt% CaO_2_ (PVP/CaO_2_ MN, needle height ≈1.65 mm), which was designed to slowly release ROS deep into infected tissue (Figure 3(C1)). In an infected porcine excision wound model, PVP/CaO_2_ MN treatment outperformed a standard silver dressing, reducing the *S. aureus* bacterial population from 4.4-log to 1.8-log CFU over five treatment days and accelerating wound closure by approximately 28% compared to *P. aeruginosa* infection and approximately 30% compared to *S. aureus* infection relative to untreated controls, while cell proliferation assays confirmed that the oxygen generated by CaO_2_ decomposition enhanced epithelial cell viability [29]. A related design was reported by Shen et al., who encapsulated CaO_2_ NPs within a dissolving MN patch (CaO_2_ NPs@MN) intended to puncture the biofilm barrier before releasing CaO_2_ for hydrolysis into O_2_ and H_2_O_2_ at the wound site. The in vitro studies confirmed the intended dual oxygen-delivery and antibacterial action, and in a diabetic wound model, the patch reduced the bacterial burden, lowered local inflammation, promoted collagen deposition, and stimulated angiogenesis, which translated into accelerated wound closure [30]. Although these studies demonstrate the therapeutic potential of CaO_2_-loaded MNs, the contribution of MN-mediated delivery should be distinguished from the intrinsic oxygen and H_2_O_2_-generating activity of CaO_2_. In the absence of an equivalent non-MN CaO_2_ formulation, improved antibacterial activity or wound closure cannot be attributed specifically to the MN architecture.

Unlike the externally triggered or substrate-coupled strategies, peroxide-based MNs rely entirely on passive hydrolysis kinetics set at the formulation stage, meaning that once the patch contacts the wound fluid, H_2_O_2_ release occurs continuously and cannot be modulated in situ. This results in a translational liability since batch-to-batch variation in CaO_2_ particle size or hydrolysis rate could shift the delivered H_2_O_2_ flux outside the therapeutic window.

#### 4.2.2. Photosensitizers

Photosensitizer-based MN systems generate ROS through light activation. Upon absorbing photons of a specific wavelength, typically in the red or near-infrared range for tissue penetration, the photosensitizer is excited to a triplet state and transfers energy either directly to molecular oxygen to generate ^1^O_2_ (Type II photodynamic therapy) or to a substrate to generate radical species such as O_2_•^−^ and •OH (Type I photodynamic therapy) [191]. Because photodynamic therapy does not depend on a specific molecular target, it circumvents the resistance mechanisms that undermine conventional antibiotics, making it well suited to multidrug-resistant biofilm infections in chronic wounds [192]. Its principal limitation, the elevated glutathione levels within biofilms that can quench photogenerated ROS before they reach bactericidal concentrations, has motivated MN designs that pair the photosensitizer with a glutathione-depleting or cascade-amplifying co-agent [193].

Yang et al. addressed this limitation with a dopamine-coated hybrid nanoparticle (SeC@PA) combining selenium NPs with the photosensitizer chlorin e6 (Ce6) and a surface layer of L-arginine, which were loaded into a dissolving MN patch. SeC@PA exhibits a bidirectional RNS-generating and RNS-scavenging functionality that is spatially regulated by the local GSH environment. It promotes reactive species generation within the biofilm while exerting a scavenging effect in the underlying wound tissue, with this functional inversion driven by the approximately ninefold difference in GSH levels between the wound covering and the underlying wound tissue [124]. Quantification by crystal violet staining attributed 18.5% of the anti-biofilm effect to ^1^O_2_, 37.6% to RNS, and 30.2% to •OH, and the treatment reduced the number of biofilm colony-forming units by 4, 3, and 1 order of magnitude relative to the control, selenium-only, and Ce6-only groups, respectively. In a biofilm-infected diabetic mouse wound, MNs produced a healing rate exceeding 95% by day 16, compared to 22%, 16%, and 15% residual wound areas in the control, Ce6-only, and selenium-only groups. Xie et al. designed a dissolving MN patch embedding a benzoxaborole-functionalized photosensitizer (TPI-BOB) that couples bacteria-selective binding, provided by the benzoxaborole moiety, with a pyridine-based cationic group that strengthens electrostatic engagement with the bacterial surface. This targeting strategy concentrated the photosensitizer at the pathogen before light activation, yielding localized photodynamic antimicrobial therapy with pronounced selectivity for Gram-positive multidrug-resistant biofilms, including those containing MRSA, and promoting closure of infected diabetic wounds [144]. A complementary multistage design was reported by Jia et al., who separated the generating and scavenging functions into distinct compartments of a single patch. A hyaluronic acid base loaded with the photosensitizer methylene blue releases rapidly on contact with tissue fluid and penetrates the biofilm to deliver antibacterial photodynamic therapy. In parallel, GelMA tips containing carbon dots and the microalga *Chlorella pyrenoidosa* address the subsequent inflammatory and hypoxic phases, with the carbon dots scavenging ROS and the microalga generating oxygen through photosynthesis. The carbon dots additionally convert 500 to 600 nm light into the 600 to 700 nm band usable by the alga to boost oxygen output, and the GelMA matrix physically separates methylene blue from the carbon dots so that scavenging does not quench the photodynamic effect, achieving spatiotemporal coordination of ROS generation, ROS scavenging, and oxygenation for chronically infected wounds [145]. In this design, the MN architecture contributes more than simple cargo carriage because physical compartmentalization separates the ROS-generating and ROS-scavenging components and enables their sequential action. This represents a platform-level function that would not necessarily be reproduced by simultaneous topical administration of the same agents.

Although photosensitizers offer an advantage in resisting bacterial multi-drug resistance, their shortcomings should also be considered. For example, photosensitizers delivered via MNs could diffuse into healthy tissue and may cause phototoxic reactions upon light exposure [194]. Another disadvantage is that wound tissue or skin pigments could attenuate incident light, hindering photosensitizer activation and reducing therapeutic performance [195]. Taken together, these limitations indicate that the clinical utility of photosensitizer-based MNs depends as much on precise light delivery and patient-specific tissue optics as on the photochemical properties of the agent itself.

#### 4.2.3. Sonodynamic Agents

Sonodynamic therapy uses ultrasound waves to excite a sonosensitizer to generate ROS through acoustic cavitation and associated redox reactions [196]. Its defining advantage over photosensitizers is penetration depth, since ultrasound reaches several centimeters into tissue whereas light penetrates only millimeters, allowing ROS generation in deep or biofilm-laden wound beds while retaining the temporal control of external activation [197,198]. Pairing a sonosensitizer with an MN solves the delivery problem, depositing the agent beneath the biofilm so that ultrasound can trigger localized ROS generation at depth.

Ouyang et al. delivered crystal-phase-engineered anatase–brookite TiO_2_ from a dissolving hyaluronic acid MN, exploiting a type II homojunction that lowers the O_2_ activation energy and improves interfacial charge transfer to yield more ROS than other TiO_2_ phases. After 15 min of ultrasound treatment, the platform achieved 99.94% antibacterial efficiency against *S. aureus*, with ultrasonic cavitation accelerating needle dissolution to disperse the sonosensitizer through deep-layered biofilm for noninvasive eradication [146]. Liang et al. extended this design by engineering ultrasmall copper peroxide nanoclusters onto oxygen-vacancy-rich TiO_2_ nanosheets (CuO_2_/TiO_2_) within an MN, coupling Fenton-based chemodynamic activity to the sonosensitizer while narrowing the TiO_2_ bandgap and supplying self-generated H_2_O_2_ and O_2_ to relieve the hypoxia that limits SDT, eliminating more than 99.99% of multidrug-resistant pathogens within 5 min and accelerating closure of MRSA-infected wounds [147]. Wang et al. instead built a core–shell MN (CCA&Lut@MN) whose hyaluronic acid shell carries L-arginine-modified copper–cysteine NPs as the sonosensitizer over a ROS-responsive luteolin core. Under ultrasound, the NPs generate •OH and ^1^O_2_ while the same ROS converts surface L-arginine into nitric oxide in a concentration-dependent manner so that oxidative and nitrative stress act together to decrease MRSA and *E. coli* survival to 4.7% and 4.47% and clear over 90% of mature biofilms, after which the responsive core degrades in the elevated-ROS environment to reduce oxidative stress and promote M2 polarization during repair in an infected diabetic wound [148].

Incorporating sonodynamic agents into MNs is an innovative strategy for chronic wound healing, but it carries notable drawbacks. Because agents such as TiO_2_- and porphyrin-based sonosensitizers consume local oxygen to generate ROS, their activity can induce microenvironmental hypoxia [199]. The resulting ROS burst can also inflict collateral damage on surrounding healthy cells, risking hyperinflammation and delaying the transition from the inflammatory to proliferative phase [200]. Finally, non-biodegradable inorganic sonosensitizers face uncertain long-term clearance and their residual accumulation within the wound bed can provoke chronic foreign-body reactions [201,202].

#### 4.2.4. Chemodynamic Agents

Chemodynamic therapy generates ROS through Fenton or Fenton-like catalysis, in which a transition-metal catalyst, most often iron or copper, converts the H_2_O_2_ present in the inflammatory wound bed into the highly cytotoxic •OH [203]. Its appeal for treating chronic wounds lies in its independence from any external trigger and its exploitation of an endogenous substrate, though its efficiency is constrained by the local availability of H_2_O_2_, Fenton ions, and the mildly acidic pH the reaction requires [204]. MN delivery addresses the substrate problem directly by pairing the metal catalyst with glucose oxidase, which oxidizes the abundant wound glucose levels to generate H_2_O_2_ and gluconic acid, simultaneously feeding the Fenton reaction and lowering the local pH into its optimal range [205].

Sun et al. built a dissolvable nanozyme MN (Fe_2_C/GOx@MNs) that releases iron carbide (Fe_2_C) NPs together with glucose oxidase into the active regions of the biofilm. The platform achieved efficient biofilm elimination and reinfection prevention in an MRSA-biofilm-infected diabetic mouse wound [149]. You et al. developed a double-layer GelMA MN that carries a ferroferric oxide/MXene (Fe_3_O_4_/MXene) heterojunction in the tips, engineered with iron vacancies that accelerate the release of Fe^2+^ and Fe^3+^ ions. These ions enter bacterial cells and trigger ferroptosis, raising intracellular ROS and malondialdehyde while depleting glutathione. Under near-infrared irradiation, the platform raised the dead-cell ratio of *E. coli* to 85.65% compared to 5.65% for the non-irradiated MN, comparable to ampicillin, whereas the same heterojunction simultaneously scavenges extracellular ROS through pH-dependent CAT and SOD-like activity, lowering the fraction of ROS-positive host cells under H_2_O_2_ challenge to 3.41% compared to 13.99% in the oxidatively stressed control. In an *S. aureus*-infected diabetic rat wound, the irradiated MN healed wounds the fastest over roughly 16 days, cleared in situ ROS, downregulated HIF-1α, and recruited M2 macrophages, so that the system raised ROS levels inside bacteria while lowering it in the surrounding tissue [150].

The two studies above illustrate similar solutions to chemodynamic therapy’s dependence on local microenvironment conditions. However, whether this condition has been overcome or substituted with a light-triggering requirement remains an unanswered question.

#### 4.2.5. Pro-Oxidant NPs

The final class of ROS-generating cargo comprises pro-oxidant NPs in which a semiconductor or defect-rich structure drives ROS production as an intrinsic material property. Most commonly, titanium dioxide and zinc oxide generate electron–hole pairs that reduce and oxidize surrounding oxygen and water into O_2_•^−^, •OH, and H_2_O_2_ [206,207]. Because these oxides in their pure form require light or ultrasound to separate charge carriers, the titanium dioxide MN systems examined in Section 4.2 are already representative of the light- and ultrasound-triggered mode. What distinguishes the platforms below is the sustained ROS output that does not depend on continuous external excitation, addressing the practical limitation that most photocatalytic and sonocatalytic NPs stop generating ROS the moment the stimulus is withdrawn.

Gong et al. addressed this directly with persistent-luminescence nanorods of copper-doped zinc germanate (Zn_2_GeO_4_:Cu, ZGC) loaded into dissolvable MNs. After a single pre-illumination treatment, the ZGC nanorods store excitation energy and release it slowly through a long persistent photocatalytic effect, continuously producing ^1^O_2_, •OH, and O_2_•^−^ radicals for at least 48 h without any need for in situ light and without the high oxygen demand that limits conventional photosensitizers. The MN tips dissolve upon insertion to deposit the nanorods directly into the biofilm, where the sustained ROS flux treats MRSA-infected wounds in a minimally invasive manner [151]. Lin et al. developed a hyaluronic acid MN (BP-CP-HA MN) carrying black phosphorus nanosheets and copper peroxide nanodots, in which the copper peroxide supplies self-generated H_2_O_2_ for chemodynamic •OH production while the black phosphorus adds light-triggered photodynamic ROS. As a result, the two together achieve ROS-amplified eradication of MRSA biofilms before the black phosphorus degrades into phosphate to support later tissue regeneration (Figure 3(A1)) [152].

Collectively, pro-oxidant NPs extend ROS generation beyond the moment of stimulation by storing excitation energy for persistent photocatalysis or by pairing a self-supplying metal peroxide. However, this condition leaves the delivered ROS governed almost entirely by the initial NP loading and activation conditions rather than being able to adjust the output once the treatment has begun.

### 4.3. Material and Mechanistic Design Strategies Beyond Functional Classification

Previous sections classify MN platforms by their functional redox outcome, whereas MOF-based architectures concern the structural platform and stimuli-responsive systems are classified by their cargo release trigger. However, these two design strategies are not mutually exclusive with each other: MOF-based systems are frequently designed with stimuli-responsive release and the MOF can itself have ROS-scavenging or generating properties. They are grouped in this section because they represent design strategies layered onto the scavenging/generating functional distinction established above and the relevant literature frequently discusses them together.

#### 4.3.1. MOF-Based MN Systems

MOFs constitute a distinct structural approach to redox-active MNs. Most MOFs are stable under neutral conditions but can undergo structural degradation in the mildly acidic environment of infected wounds. This pH responsiveness can be exploited to promote cargo release preferentially at sites of pathological acidity [208]. Incorporating MOFs into an MN matrix may mitigate some limitations associated with direct administration of particulate MOF systems, including limited localization and premature exposure to the wound environment [88,209]. However, these advantages should be distinguished from the intrinsic catalytic, ion-releasing, or redox activity of the MOF itself, and direct comparisons with equivalent non-MN MOF formulations remain necessary to establish the magnitude of the MN-specific contribution [210].

The magnesium organic framework MN of Yin et al., examined in Section 4.1.3, illustrates the redox-active framework, since the Mg-MOF releases both Mg^2+^ and ROS-scavenging polyphenol gallic acid under acidic conditions directly from the framework structure, coupling antioxidation to Mg^2+^-driven angiogenesis [140]. A complementary copper-based design was reported by Yao et al., who loaded nitric oxide into the copper-benzene-1,3,5-tricarboxylate framework HKUST-1, encapsulated it in graphene oxide, and embedded the resulting microparticles in a porous poly(ethylene glycol) diacrylate MN. The graphene oxide shell provided near-infrared photothermal responsiveness, enabling on-demand control of NO release. In a diabetic rat wound, the irradiated patch achieved 99% closure within 13 days and promoted vascularization and tissue regeneration (Figure 3(B3)) [125]. Jiang et al. employed a zinc-based framework, encapsulating the marine polysaccharide fucoidan within a Zn-MOF and loading it into an MN for MRSA-infected wounds, where framework degradation released antibacterial Zn^2+^ alongside fucoidan for subcellular targeted anti-inflammatory and pro-healing action [153].

MOF-based MNs show that the framework can serve simultaneously as a high-capacity reservoir, an intrinsic source of therapeutic metal ions, and a built-in stimuli-responsive gate. Pairing MOFs with acidic or near-infrared triggers position them as a natural bridge to the stimuli-responsive systems in which such triggered release becomes the central design principle. However, MOFs are structurally sensitive to body fluids and physiological pH, so the porous framework can degrade unpredictably once inserted into the hydrated wound bed. As a result, metal ions may release at a rate that is difficult to standardize, ultimately causing oxidative stress and cytotoxicity [211].

#### 4.3.2. Stimuli-Responsive MN Systems for ROS Modulation

Stimuli-responsive MNs place cargo release under the control of pathological properties intrinsic to the chronic wound, so that the therapeutic output self-regulates in proportion to the local disease burden. Three properties dominate this field: the elevated flux of H_2_O_2_ and •OH that marks oxidative overload, the acidic or alkaline pH shifts that accompany infection and metabolic dysregulation, and the aberrant activity of enzymes such as gelatinase and hyaluronidase, which are secreted by active bacterial communities [212,213,214]. The following subsections examine each responsive modality.

Stimuli-responsive and compartmentalized systems provide some of the clearest examples of an MN-specific design contribution. In these platforms, the MN is not merely a carrier for an active compound; its spatial organization and material composition determine when and where individual cargos are released. Bilayer, core–shell, and multi-compartment architectures can physically separate otherwise incompatible therapeutic functions, while ROS-, pH-, or enzyme-responsive matrices can regulate cargo release in response to local pathological cues. Nevertheless, demonstrating responsive release from an MN does not necessarily demonstrate superior therapeutic efficacy over an equivalent responsive non-MN formulation. Direct head-to-head comparisons remain necessary to quantify the added therapeutic value attributable specifically to the MN architecture.

##### ROS-Responsive Systems

ROS-responsive MNs exploit the elevated H_2_O_2_ and •OH of the chronic wound as the trigger itself, most commonly through oxidation-sensitive covalent linkers built into the needle matrix. Phenylboronate ester bonds, formed between phenylboronic acid moieties and diol-containing polymers, are among the most widely used ROS-responsive linkages because they undergo oxidative cleavage in the presence of H_2_O_2_, promoting cargo release while simultaneously consuming the oxidant. Thioketal and other oxidation-labile groups can serve the same function [215,216]. Since release scales with the local ROS concentration, these systems deliver antioxidant or anti-inflammatory payloads into the most oxidatively stressed regions and diminish as balance is restored.

Zhang et al. built a double-layer patch (MN@DOX+RES, 225 tips, 800 μm high) whose rapidly dissolving hyaluronic acid backing releases doxycycline for an early antibacterial burst, while ROS-responsive tips of a boronate-ester network (3-aminophenylboronic-acid-grafted aldehyde-modified hyaluronic acid crosslinked with PVA) encapsulate resveratrol inclusion complexes. The patch scavenged over 70% of DPPH and about 80% of ABTS radicals and shifted macrophages from M1 toward M2 via the Keap1/Nrf2/HO-1 pathway. In an *S. aureus*-infected full-thickness rat wound, it reached 89.9% closure by day 7 compared to 50.3% for controls, leaving 2.1% by day 10, and showed the highest collagen deposition and vessel density among all groups (Figure 3(A3)) [154]. Ultimately, the study illustrates that combining spatiotemporally staged release of antibacterial, antioxidant and anti-inflammatory compounds within a single MN platform is a promising strategy and, with further validation, could develop into a translationally viable approach.

##### pH-Responsive Systems

The infected chronic wound carries a characteristic pH signature since bacterial metabolism and biofilm formation drive the local environment acidic while unhealed diabetic wounds often shift to be weakly alkaline; thus, deviation from the mildly acidic pH of healthy skin can serve as a release trigger [217,218]. pH-responsive MNs exploit this through acid-dependent chemistry, most often imine or Schiff-base bonds and boronate esters that hydrolyze as pH drops, or MOFs whose coordination bonds are stable at neutral pH but dissolve under the acidity of an infected wound bed [219,220,221].

Xue et al. developed a pH-responsive hydrogel MN (LSI-GCA) that loads isoliquiritigenin into stearic-acid-modified Lycium barbarum polysaccharide micelles crosslinked with GelMA in the tips, backed by a chitosan–anthocyanin layer that changes color for real-time pH monitoring. Isoliquiritigenin release accelerates under the alkaline wound conditions, and the released drug scavenges ROS and suppresses inflammation by activating the NRF2 pathway. Furthermore, in a diabetic mouse model, the patch promoted angiogenesis, collagen deposition, and M2 macrophage polarization while accelerating wound closure [155]. The system illustrates the pairing of pH sensing with pH-gated antioxidant delivery in a single MN, a dual sensing and treating capability that few of the platforms discussed in this review achieve simultaneously.

##### Enzyme-Responsive Systems

Enzyme-responsive MNs use the aberrant enzymatic activity of the infected wound as the release trigger, exploiting the fact that active bacterial communities and dysregulated host tissue secrete specific enzymes at elevated levels. Bacterial hyaluronidase, which degrades hyaluronic acid and is secreted by pathogens such as *S. aureus* to spread through host tissue, and MMPs, particularly the gelatinases MMP-2 and MMP-9, which are overexpressed in the chronic inflammatory wound, are the most commonly studied classes [213,222]. Building the needle matrix or a cargo linker from a substrate of one of these enzymes makes the release self-targeting since the payload is liberated in proportion to local enzymatic activity, which is highest where infection and inflammation are most active [223].

A gelatinase-triggered design was reported by the multifaceted MN patch of Xin et al., which combines a bromothymol blue pH visual-warning layer with a GelMA matrix that releases mesenchymal-stem-cell-derived exosomes in response to pathological gelatinase, delivering triple payloads for hemostasis, antibacterial and anti-inflammatory action, and scar reduction, and achieving over 95% clearance of MRSA and imipenem-resistant *P. aeruginosa* with biofilm inhibition and disruption rates above 90% (Figure 3(A2,B1)) [156]. The gelatinase dependence ensures that exosome release is timed to the phase with the most active proteolytic inflammation. Ultimately, enzyme-responsive triggering offers a specificity advantage since gelatinase activity tracks active pathogenic or proteolytic processes more directly than a bulk chemical shift.

##### Multi-Stimuli-Responsive Systems

The chronic wound rarely presents a single pathological sign in isolation since elevated ROS, acidic or alkaline pH shifts, and abnormal enzyme activity coexist and work together across the healing phases. Multi-stimuli responsive MNs exploit this overlap by building sensing elements for two or more cues into a single platform, so that release is gated only when several disease signals coincide, sharpening spatial and temporal targeting beyond what any single-trigger design achieves [127,224]. The most integrative of these platforms go further, coupling a generating function to a scavenging one so that the same MN switches its redox output according to whether the local cue signals active infection or entry into repair [225].

A dual-triggered example was reported by Diao et al., whose PCMA MN patch combines a pH and ROS dual-responsive hydrogel matrix, gallic acid-grafted chitosan crosslinked with TSPBA-modified PVA, mangiferin-loaded PLGA microspheres and an antibacterial silver–gallic acid metal–phenolic coating. The coating delivers an initial Ag+ burst for rapid biofilm disruption, a mechanism independent of ROS generation, after which the acidic and oxidative properties of the infected wound bed break the TSPBA boronate crosslinks to release mangiferin in a pH- and ROS-responsive manner, promoting angiogenesis and shifting macrophages toward the reparative M2 phenotype. This spatiotemporal cascade accelerated wound closure by 14.25% compared to conventional antimicrobial therapy, coordinating rapid pathogen killing with sustained pro-regenerative immunomodulation [128].

Yang et al. developed a detachable core–shell pH/ROS-responsive MN (EM/Man MNs, 10 by 10 array, 600 μm needle height) pairing a quaternary ammonium chitosan shell for penetration and bacterial capture with a ROS-degradable PVA-TSPBA core loading mannose-modified NPs that co-deliver epigallocatechin-3-gallate and metformin. Cargo release accelerated with both rising H_2_O_2_ and decreasing pH, and the released NPs cascade-target macrophage mitochondria, scavenging mitochondrial ROS and activating the AMPK/SIRT1/PGC-1alpha axis to reprogram macrophages toward M2. The patch reached about 90% antibacterial efficiency and reduced the residual wound area to 1% by day 15 compared to 37.33% for controls while suppressing hypertrophic scarring in a rabbit-ear model [157].

Taken together, these two studies illustrate that multi-stimuli gating and mitochondria- or macrophage-targeted redox modulation can be layered onto a single MN. However, whether requiring coincident cues improves targeting over the simpler responsive designs remains to be tested. More broadly, the studies reviewed in this section indicate a shift from constitutively active redox formulations toward platforms in which ROS modulation is controlled by spatial compartmentalization, pathological cues, or external activation.

## 5. Challenges and Limitations

Despite the mechanistic advantages, the translational record of redox-active MNs in chronic wound healing is effectively empty. Only a single MN product has completed clinical evaluation for a wound indication, and it carries no redox-active function [226]. However, MN devices that do carry regulatory clearance are mechanical microneedling instruments indicated for facial acne scarring and rhytides, which deliver no payload and possess no redox function. The commercial history of MN drug–device combination products shows that successful clinical results do not ensure reliability in the market. The intradermal influenza vaccine delivered by hollow microinjection reached the market but was subsequently withdrawn, driven by constrained manufacturing scalability and the absence of economies of scale rather than by any deficiency in immunogenicity, illustrating that demonstrated clinical efficacy is insufficient for sustained commercialization [227]. More relevant is the case of the zolmitriptan-coated MN array patch for acute migraine, which failed to secure approval because of manufacturing-driven pharmacokinetic variability, with regulatory concern centered on inconsistent systemic exposure rather than therapeutic effect [227]. For redox-active platforms, the demands on dose uniformity are considerably more important than for a coated small molecule. Progress to overcome these limitations will require the field to shift emphasis from the demonstration of new chemistries toward the characterization of existing ones under conditions that approximate clinical use, including repeated application, extended follow-up, and manufacturing at a scale where variability becomes measurable.

## 6. Conclusions and Future Directions

Section 4 highlights a principle the field has not yet stated explicitly. Some of the most effective platforms scavenge and generate ROS at the same time. The SeC@PA system of Yang et al. produces a reactive species inside the biofilm while scavenging in the tissue underneath. The inversion is driven by a ninefold difference in GSH between the wound covering and the underlying wound tissue. You et al. achieve the same split by cellular compartment, raising ROS inside bacteria, resulting in 85.65% killing, while lowering the ROS-positive host cell fraction to 3.41%. Jia et al. achieved it by physically separating the two functions within the patch. In these systems, the spatial separation arises from material chemistry or biological context rather than a defined design parameter. Deliberately engineering such spatial partitioning and characterizing the resulting ROS gradient could therefore transform an observed material behavior into a controllable design feature and provide a stronger mechanistic basis for future translational studies.

Translation will depend not only on therapeutic mechanisms but also on reproducible control of material properties. The performance of these platforms is influenced by properties that are not captured by standard content-uniformity. Xuan et al. make the point clearly: controlling the Ce^3+^/Ce^4+^ surface ratio alone raised H_2_O_2_ decomposition from 56.37% to 89.42%. Comparing across studies is difficult for a related reason. Antioxidant capacity is reported as percentage DPPH scavenged, percentage ABTS scavenged, percentage H_2_O_2_ decomposed, or enzyme-equivalent units, and assay conditions are rarely given in full. This is particularly relevant because these materials are strongly pH-dependent and the chronic wound microenvironment can differ substantially from physiological pH. The MXene-TA/Fe system of Li et al. performs best between pH 7.5 and 9.5, so a measurement at physiological pH may not fully capture its activity under wound-relevant conditions.

The preclinical evidence also requires disease and tissue relevance. Almost all efficacy data presented here come from rodent excisional wounds. These wounds heal mainly by closure rather than re-epithelialization, carry no established biofilm, and lack the protease excess of a human chronic wound. Furthermore, the insertion and fracture data behind the geometric rules all come from intact skin or its surrogates, yet these devices are justified on the grounds that they penetrate necrotic tissue, eschar, and biofilm. Agarose or gelatin substrates covered with a defined thickness of simulated slough or established biofilm would allow the penetration threshold and insertion depth to be measured under realistic conditions. Porcine and biofilm-infected models under repeated application would give efficacy data that actually predicts performance in a human ulcer.

As a result, translation remains a major gap in the field. No redox-active MN has entered clinical evaluation for chronic wounds, and the products holding regulatory clearance are mechanical microneedling instruments carrying neither payload nor redox function. The commercial record of MN combination products suggests that manufacturing consistency is what determines which platforms reach patients. Redox-active MNs remain a well-reasoned answer to a condition for which current therapy is largely supportive, and whether they become more than that depends on how carefully the existing platforms are characterized.

## Figures and Tables

**Table 2:** Redox-active MN platforms discussed in this review, classified by therapeutic direction, redox-active component, and mode of activation. Platforms marked bidirectional exert scavenging and generating functions concurrently, partitioned by location, type and geometry, compartment, or healing phase (porous non-dissolving polymer MNs with embedded cargo were grouped under solid).

Platform	MN Type and Geometry	Direction	Redox Component	Activation	Reference
MSN@Met-CeO_2_	Swelling hydrogel-forming MN, conical shape, 600 μm tip height	Scavenging	CeO_2_ nanozyme	ROS-responsive matrix	[129]
Lys-AFs-Ceria	Dissolving MN, conical shape, 600 μm tip height	Scavenging	Templated CeO_2_ nanozyme	Passive, GOx cascade	[130]
CuTA@MnO_2_	Dissolving MN (slowly biodegradable, NIR-accelerated release), conical shape, 300 μm tip height	Scavenging	MnO_2_ nanozyme	NIR photothermal	[131]
Dual-Layer MnO_2_/NO	Swelling hydrogel-forming MN, conical shape, tip height not reported	Scavenging	MnO_2_ nanozyme	Passive, layered	[132]
ALA@CFP	Swelling hydrogel-forming MN, square pyramid shape, 400 μm tip height	Bidirectional	Co-Fe Prussian blue	Light, 5-ALA	[133]
SilMA CaO_2_/CAT	Hydrogel-forming MN (crosslinked SilMA hydrogel; swelling not characterized), conical shape, 600 μm tip height	Scavenging	CAT with CaO_2_	Passive	[134]
GelMA CaO_2_/CAT	Swelling hydrogel-forming MN, conical shape, tip height not reported	Scavenging	CAT with CaO_2_	Passive	[135]
CAT-(MnSH)x@PMB	Dissolving MN, conical shape, ~800 μm tip height	Scavenging	CAT-templated Mn nanozyme	Passive	[136]
GelMA curcumin/ZnO	Swelling hydrogel-forming MN, square pyramid shape, 545 ± 15 μm tip height	Scavenging	Curcumin	Passive, PLGA depot	[137]
Nb_2_C/curcumin	Hydrogel-forming MN (UV crosslinked hydrogel; swelling not characterized), conical shape, 600 μm tip height	Scavenging	Curcumin	NIR photothermal	[138]
MXene-TA/Fe	Hydrogel-forming MN (UV crosslinked hydrogel; swelling not characterized), conical shape, 500 μm tip height	Scavenging	Tannic acid metal–phenolic	NIR photothermal	[139]
Mg-MOF/GO-Ag	Dissolving MN, square pyramid shape, 500 μm tip height	Scavenging	Gallic acid in Mg-MOF	pH-responsive	[140]
CINP@SiO_2_-HA	Dissolving MN, conical shape, 600 μm tip height	Scavenging	Cuttlefish melanin	NIR photothermal	[141]
PDA Core–Shell	Dissolving MN, square pyramid shape, 860 μm tip height	Scavenging	Polydopamine	Passive, shell degradation	[142]
Se-CQD/Astilbin	Swelling hydrogel-forming MN, conical shape, 600 μm tip height	Scavenging	Se-doped carbon dots	Passive, sequential bilayer	[143]
PVP/CaO_2_	Dissolving MN, conical shape, 1700 μm tip height	Generating	CaO_2_	Passive hydrolysis	[29]
CaO_2_ NPs@MN	Dissolving MN, conical shape, 1790 ± 30 μm tip height	Generating	CaO_2_	Passive hydrolysis	[30]
SeC@PA	Dissolving MN, conical shape, tip height not reported	Bidirectional	Ce6 photosensitizer, Se NP	Light, GSH gradient	[124]
TPI-BOB	Dissolving MN	Generating	Benzoxaborole photosensitizer	Light	[144]
MB/CD/Chlorella	Dissolving MN	Bidirectional	Methylene blue, carbon dots	Light, compartmentalized	[145]
Anatase–brookite TiO_2_	Dissolving MN (ultrasound-accelerated dissolution)	Generating	TiO_2_ sonosensitizer	Ultrasound	[146]
CuO_2_/TiO_2_	Dissolving MN, conical shape, 600 µm tip height	Generating	CuO_2_, TiO_2_	Ultrasound	[147]
CCA&Lut@MN	Dissolving MN (core–shell: soluble HA shell; degradable hydrogel core), conical shape, 600 µm tip height	Bidirectional	Cu–cysteine, luteolin	Ultrasound, then ROS	[148]
Fe_2_C/GOx@MNs	Dissolving MN	Generating	Fe_2_C Fenton catalyst	Endogenous substrate	[149]
Fe_3_O_4_/MXene	Swelling hydrogel-forming MN, conical shape, tip height not reported	Bidirectional	Fe_3_O_4_ heterojunction	NIR, cellular partition	[150]
Zn_2_GeO_4_:Cu	Dissolving MNs	Generating	Persistent-luminescence oxide	Pre-illumination only	[151]
BP-CP-HA	Dissolving MN, conical shape, 600 µm tip height	Generating	Black phosphorus, CuO_2_	Light	[152]
NO@HKUST-1@GO	Solid MN (porous, non-dissolving), shape and tip height not reported	Generating	Cu-MOF, NO donor	NIR photothermal	[125]
Zn-MOF/Fucoidan	Dissolving MN, square pyramid shape, 650 µm tip height	Generating	Zn-MOF	pH-responsive	[153]
MN@DOX+RES	Swelling hydrogel-forming MN, square pyramid shape, 800 µm tip height	Scavenging	Resveratrol	ROS-responsive	[154]
LSI-GCA	Hydrogel-forming MN (UV crosslinked GelMA tips; porous; swelling not characterized), conical shape, tip height not reported	Scavenging	Isoliquiritigenin	pH-responsive, alkaline	[155]
Exosome/BTB	Swelling hydrogel-forming MN, conical shape, 600 µm tip height	Scavenging	MSC exosomes	Gelatinase-responsive	[156]
PCMA	Hydrogel-forming MN (slowly degradable, pH/ROS-accelerated; swelling not characterized), conical shape, tip height not reported	Bidirectional	Gallic acid, mangiferin	pH and ROS	[128]
EM/Man MNs	Dissolving MN (detachable core–shell), conical shape, 600 µm tip height	Scavenging	EGCG, metformin	pH and ROS	[157]

## Data Availability

No new data were created or analyzed in this study. Data sharing is not applicable to this article.

## Abbreviations

The following abbreviations are used in this manuscript:

ROS	Reactive oxygen species
RNS	Reactive nitrogen species
GSH	Glutathione
MN	Microneedle
SOD	Superoxide dismutase
MPO	Myeloperoxidase
AGEs	Advanced glycation end products
CAT	Catalase
MMP	Metalloproteinase
ECM	Extracellular matrix
PLA	Poly-lactic acid
PVP	Polyvinylpyrrolidone
PVA	Polyvinyl alcohol
CMC	Carboxymethyl cellulose
HFMNs	Swelling hydrogel-forming microneedles
PMVE/MA	Poly(methyl vinyl ether-co-maleic acid)
PEGDA	Poly(ethylene glycol) diacrylate
GelMA	Gelatin methacryloyl
MeHA	Methacrylated hyaluronic acid
PDMS	Polydimethylsiloxane
MOF	Metal–organic framework
POD	Peroxidase
SilMA	Silk fibroin methacryloyl
Nb2C	Niobium carbide
PLGA	Poly lactic-co-glycolic acid
GPx	Glutathione peroxidase
Se-CQDs	Selenium-doped carbon quantum dots
NPs	Nanoparticles
